# Mean generation function model in AIDS epidemic estimation

**DOI:** 10.1186/s12911-022-01825-6

**Published:** 2022-04-16

**Authors:** Lei Yuan, Shiyin Tian, Zhe Zhao, Pei Liu, Lijuan Liu, Jinhai Sun

**Affiliations:** 1 Department of Health Management, Faculty of Health Service, Second Military Medical University, Naval Medical University, No. 800 Xiangyin Road, Shanghai, 200433 People’s Republic of China; 2 Department of Hospital Administration, Changzheng Hospital, Naval Medical University, Shanghai, 200003 China; 3 Department of Mathematics and Physics, Faculty of Health Service, Naval Medical University, Shanghai, 200433 China

**Keywords:** AIDS, Incidence, Mortality, Mean generation function model, Forecast, China

## Abstract

**Background:**

Since the first case of HIV infection was reported in China in 1985, the incidence and mortality of AIDS have been increasing rapidly, which has caused serious damage to the life and health of people in China and all over the world. Therefore, it is of great significance to study the technique for predicting AIDS morbidity and mortality. The purpose of this research is to explore the applicability of the mean generation function model (MGFM) in the early warning of AIDS morbidity and mortality, to predict its prevalence trend, to enrich the prediction techniques and methods of AIDS research and to provide suggestions for AIDS transmission control.

**Methods:**

In this research, the MGFM was applied to predict the incidence and mortality of AIDS in China. AIDS incidence and mortality data in China from 2008 to 2019 were used to construct the prediction model.

**Results:**

The MGFM can predict the annual incidence and mortality of AIDS. The model constructed in this research predicted that the incidence and mortality of AIDS in China will continue to increase from 2020 to 2023.

**Conclusion:**

The mean birth function model was an effective method to monitor and predict the changing trend of AIDS incidence and mortality in China.

**Supplementary Information:**

The online version contains supplementary material available at 10.1186/s12911-022-01825-6.

## Background

Human immunodeficiency virus (HIV) is a virus that attacks the human immune system. HIV destroys CD4 cells and weakens people's immunity to opportunistic infections. Acquired immunodeficiency syndrome (AIDS) is the latest stage of HIV infection [1]. At present, HIV is still a major global public health problem, and there is no permanent cure [2, 3]. Since the first AIDS patient was diagnosed in 1981, AIDS has exploded exponentially worldwide [4]. The WHO's *Global progress report on HIV, viral hepatitis and sexually transmitted infections, 2021* shows that approximately 1.5 million people were newly infected with HIV in 2020, and 680,000 people died from HIV-related diseases. By 2020, there were 37.7 million HIV-infected people in the world [5]. Especially after the outbreak of COVID-19 in 2020, AIDS patients were extremely vulnerable to infection due to their low immunity, which made the prevention and treatment of AIDS more difficult [6–9].

In China, AIDS is classified as a Class B infectious disease. According to the *General Situation of National Statutory Infectious Diseases in 2020* published by the Disease Control and Prevention Bureau of the National Health Commission, there were 62,167 new AIDS patients and 18,819 deaths from all causes of AIDS in 2020. AIDS has become an infectious disease with the sixth highest incidence rate and the highest mortality rate of Class A and B infectious diseases in China [10]. As of October 2019, 958,000 Chinese citizens were living with the infection [11]. To effectively prevent and control the spread of AIDS, the Chinese government formulated the *Implementation Plan for Preventing the Spread of AIDS *(*2019–2022*). It has promoted six major projects to curb the spread of AIDS, such as the AIDS prevention publicity and education project, the AIDS comprehensive intervention project, the AIDS expanded detection and treatment project, the AIDS prevention comprehensive social management project, the elimination of mother-to-child transmission of AIDS and the AIDS prevention education project for students.

Using mathematical models to predict the epidemic trend of AIDS can provide a basis for better prevention and control of the spread of AIDS. To date, many scholars have used various mathematical models to predict the changing trend of AIDS incidence and mortality, such as the ARIMA model; this model was used to study the epidemic trend of AIDS and HIV in China, but the ARIMA model is an autocorrelation method. The ability to predict random frequent bursts is limited [12]. Studies in hydrometeorology showed that, compared to the ARIMA model, the time series MGFM can better fit the change trend of time series, grasp the extreme value well, and can better predict the long-term change trend. This method has been widely used in the forecast work of hydrometeorology in China [13, 14]. However, according to our search, there has been no report on the application of MGF in disease prediction in the field of public health.

Therefore, this study introduced the MGFM to explore the applicability of the model in AIDS disease prediction to enrich AIDS prediction methods and provide reference suggestions for AIDS prevention and control in China.

## Methods

### Data source

In this research, we used AIDS incidence and mortality data from 2008 to 2019 collected in 31 provinces (excluding Hong Kong, Macao and Taiwan) and published in the *China Health Statistics Yearbook* compiled by China National Health Commission [15]. The data are released annually after being reviewed by the Chinese CDC. The monitored population covers 31 provinces in mainland China, and the data collection and release process are as follows:

1. Residents voluntarily undergo HIV antibody testing at the time of medical treatment and physical examination in medical institutions.
2. Medical institutions report HIV antibody-positive residents to the local CDC, and the local CDC will conduct a diagnosis test on suspected patients.
3. The local CDC reports confirmed positive tests to the medical institutions and urges the medical institutions to report the patient data for HIV/ARDS through the Chinese disease prevention and control information system.
4. The Center for STD/AIDS Prevention and Control of China CDC uses the China Information System for Disease Control and Prevention to collect and publish HIV/AIDS data nationwide.

### Geographical distribution of China

According to the direction and location, Chinese geography can be divided into three regions [15], namely, eastern, central, and western. The distribution of 31 regions included in this study is as follows:

The Eastern region includes 11 provinces: Beijing, Tianjin, Hebei, Liaoning, Shanghai, Jiangsu, Zhejiang, Fujian, Shandong, Guangdong, and Hainan.

The Central region includes 8 provinces: Shanxi, Jilin, Heilongjiang, Anhui, Jiangxi, Henan, Hubei, and Hunan.

The Western region includes 12 provinces: Inner Mongolia, Chongqing, Guangxi, Sichuan, Guizhou, Yunnan, Tibet, Shaanxi, Gansu, Qinghai, Ningxia, and Xinjiang.

### Basic statistics analysis

A descriptive statistics method was used to analyse the dynamic change trend of AIDS incidence and mortality, and the time series MGFM of AIDS incidence and mortality was constructed according to the collected data. Our research used DPS 17.10 software to help us build these models [16].

### MGFM

#### Brief introduction

Time series with equal interval sample size of N:

1 $$x(t) = \{ x(1),x(2), \ldots ,x(N)\} ,$$

The mean-generating function (MGF) of each order is constructed according to the following formula:

2 $$\overline{x}_{l} (i) = \frac{1}{{n_{l} }}\sum\limits_{j = 0}^{{n_{l} - 1}} {x(i + jl)} ,$$

Among them,$i = 1,2, \ldots ,l,\;1 \le l \le M,\;n_{l} = INT(N/l),\;M = INT(N/2)$, where *N* is the sample length of the sequence,* t* is the sampling time, *l* is the time interval length of the MGFM, *M* is the maximum period length, and *INT* is rounding. Cyclic extrapolation is carried out on MGF $\overline{x}_{l} (i)$ to construct an extended series of periodic predictions:

3 $$\begin{aligned} & f_{l} (t) = \overline{x}_{l} \left[ {t - l \cdot INT\left( {\frac{t - 1}{l}} \right)} \right], \\ & t = 1,2, \ldots ,N + q, \\ \end{aligned}$$

where *q* is the prediction step, and *M* periodic function sequences of length *N* + *q* can be obtained. Taking these periodic continuation series as factors and performing stepwise regression analysis with the original series, we can establish a regression equation for simulation and prediction.

Fitting the high-frequency components of the original data $x(t)$ for differential operation, the differential operation acts as a high-pass filter:

$$\Delta x(t) = x(t + 1) - x(t),\quad t = 1,2, \ldots N - 1,$$

$$\Delta^{2} x(t) = \Delta x(t + 1) - \Delta x(t),\quad t = 1,2, \ldots N - 2,$$

The corresponding sequence is recorded as $x^{(1)} (t)$ and $x^{(2)} (t)$, and its MGFM and continuation sequence are recorded as $\overline{x}_{l}^{(1)} ,\;\overline{x}_{l}^{(2)}$ and $f_{l}^{(1)} (t),\;f_{l}^{(2)} (t)$, respectively.

To fit the upward increasing or downward decreasing trend in the original data sequence, the cumulative continuation sequence is further established:

4 $$f_{l}^{(3)} (t) = x(1) + \sum\limits_{i = 1}^{t - 1} {f_{l}^{(1)} (i + 1)} ,$$

Among them,$t = 2, \ldots ,N;\;l = 1,2, \ldots M$, $f_{l}^{(3)} (1) = x(1)$. The cumulative continuation sequence replaces the difference value of each moment with the first-order difference MGFM.

After the above process, finally, a total of 4 M continuation sequences of MGFMs are obtained:

$$f_{l} (t),f_{l}^{(1)} (t),f_{l}^{(2)} (t),f_{l}^{(3)} (t),\quad l = 1,2, \ldots M.$$

The purpose is to obtain a high-precision prediction model through screening, that is, there are more independent variables to be screened.

#### Double scoring criteria

The research results are as follows $\left[ \frac{N}{2} \right] + \left[ {N - {1 \mathord{\left/ {\vphantom {1 2}} \right. \kern-\nulldelimiterspace} 2}} \right] + \left[ {N - 1} \right]$ MGFMs; if these are all involved in modelling, this will lead to excessive calculation. Therefore, the generated MGFM is preliminarily screened by the double scoring criterion, which aims to improve the fit of the model. The double scoring criterion is defined as follows:

5 $$CSC = S_{1} + S_{2}$$

where $S_{1} = (N - k)(1 - \frac{{Q_{k} }}{{Q_{y} }})$ is the score for quantity, that is, the fine score,

$S_{2} = 2I = 2\left[ {\sum\nolimits_{i = 1}^{G} {\sum\nolimits_{j = 1}^{G} {n_{ij} \ln n_{ij} + N\ln N - \left( {\sum\nolimits_{i = 1}^{G} {n_{i} \ln n_{i} + \sum\nolimits_{j = 1}^{G} {n_{j} \ln n_{j} } } } \right)} } } \right]$ is the score for the trend, called the rough score; *N* is the sample length; *k* is the number of variables in the statistical model; and *n*_*ij*_ is the number in the contingency table of class *i* events and class *j* events.

*Q*_*k*_ is the sum of residual squares of the model, and the calculation formula is as follows:

$$Q_{k} = \frac{1}{N}\sum\limits_{t = 1}^{N} {(x(t) - \hat{x}(t))^{2} }$$.

$\hat{x}(t)$ is the prediction value.

*Q*_*y*_ is the sum of the squares of the total deviation of the model, and the calculation formula is as follows:

$$Q_{y} = \frac{1}{N}\sum\limits_{t = 1}^{N} {(x(t) - \overline{x}(t))^{2} }.$$

Type, $$\overline{x} = \frac{1}{N}\sum\limits_{t = 1}^{N} {x(t)}$$.

When the model is linear and *k* is fixed as a small quantity, $N \to \infty ,\;(N - k) \approx N \to \infty ,\;S_{1} \approx NR^{2}$ of the asymptotic distribution is $\chi_{{v_{1} }} ,\;v_{1} = k$.

*R* is a complex correlation coefficient,$v_{2} = (G - 1)(G - 1)$, according to $\chi^{2}$. The additivity of distribution is as follows:

$$\chi_{v}^{2} = \chi_{v1}^{2} + \chi_{v2}^{2} ,\;v = k + (G - 1)(G - 1)$$.

Therefore, the CSC value of the original sequence x(t) can be calculated for each MGFM as a fitting sequence when $CSC > \chi_{v}^{2}$ and the sequence is selected.

#### Selection of the optimal equation

According to the above process, the CSC values of each sequence are calculated separately. The optimal subset method is used for screening based on the CSC value; that is, all possible combinations are listed, and the regression equation with the largest CSC value is selected as the optimal equation. When the number of variables is different and the CSC value is the same, the equation with fewer variables is taken as the optimal regression equation for simple calculation.

## Results

### Basic information

**Fig. 1:**
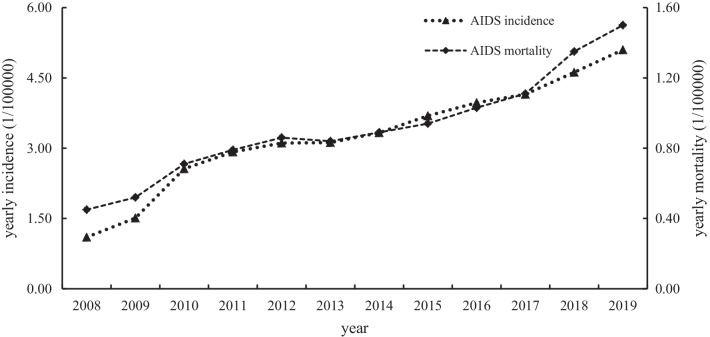
Incidence and mortality of AIDS in China from 2008 to 2019

From 2008 to 2019, the incidence and mortality of AIDS in China showed an increasing trend year by year, as shown in Fig. 1. Table 1 shows the growth rate of AIDS mortality and morbidity in China and the order of incidence and mortality of infectious diseases in that year. During this period, the average annual growth rates of AIDS morbidity and mortality were 14.96% and 11.57%, respectively. In 2019, the incidence of AIDS ranked 7th in Class A and B infectious diseases, and the mortality of AIDS ranked 1st in Class A and B infectious diseases.

**Table 1:** Changes in AIDS incidence and mortality in China from 2008 to 2019

Year	Incidence (Per 100,000 people)	The incidence of infectious diseases by year	Chain growth rate (%)	Growth rate (%)	Mortality (Per 100,000 people)	The mortality rate of infectious diseases by year	Chain growth rate (%)	Growth rate (%)
2008	1.10	12	–	–	0.45	4	–	–
2009	1.51	12	37.27	37.27	0.52	1	15.56	15.56
2010	2.56	10	69.54	132.73	0.71	3	36.54	57.78
2011	2.92	9	14.06	165.45	0.79	4	11.27	75.56
2012	3.11	7	6.51	182.73	0.86	1	8.86	91.11
2013	3.12	7	0.32	183.64	0.84	1	-2.33	86.67
2014	3.33	10	6.73	202.73	0.89	1	5.95	97.78
2015	3.69	8	10.81	235.45	0.94	1	5.62	108.89
2016	3.97	7	7.59	260.91	1.03	1	9.57	128.89
2017	4.15	7	4.53	277.27	1.11	1	7.77	146.67
2018	4.62	7	11.33	320.00	1.35	1	21.62	200.00
2019	5.10	7	10.39	363.64	1.50	1	11.11	233.33

### Geographical distribution of AIDS incidence and mortality in China in 2019

**Fig. 2:**
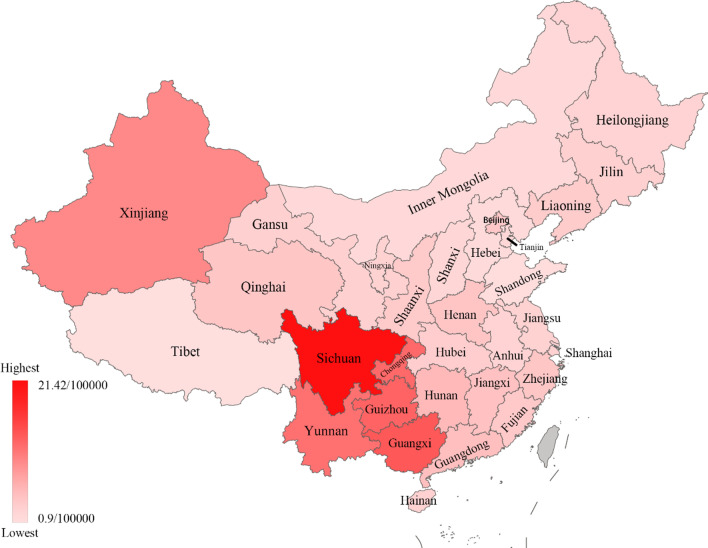
Geographical distribution of AIDS incidence in 31 provinces of China in 2019

**Fig. 3:**
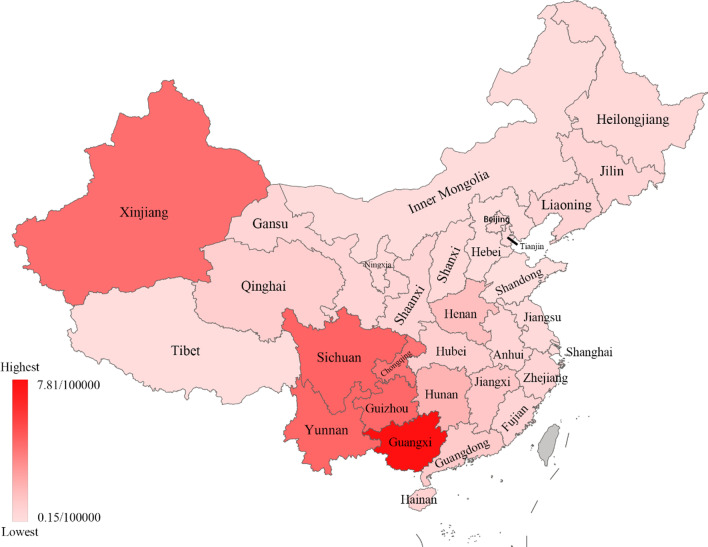
Geographical distribution of AIDS mortality in 31 provinces of China in 2019

According to the public data of official Chinese departments, Table 2 shows the specific numbers of AIDS incidence and mortality in 31 provinces and regions in China (excluding Hong Kong, Macao, and Taiwan) in 2019 and ranks them from largest to smallest. Figure 2 shows the geographical distribution of AIDS incidence in China, and Fig. 3 shows the geographical distribution of AIDS mortality in China. From the two figures, we can see that the incidence and mortality of AIDS in western China are relatively high.

**Table 2:** AIDS cases in various provinces (municipalities and autonomous regions) in 2019^a^ (1/100,000)

Region	Incidence rate	Incidence rank	Mortality rate	Mortality rank
Beijing	3.17	13	0.37	24
Tianjin	1.58	27	0.31	26
Hebei	1.31	29	0.18	30
Shanxi	1.65	26	0.38	23
Inner Mongolia	1.37	28	0.21	28
Liaoning	2.70	16	0.51	17
Jilin	2.28	19	0.48	20
Heilongjiang	1.96	25	0.38	22
Shanghai	2.21	20	0.21	27
Jiangsu	2.07	23	0.31	25
Zhejiang	3.30	12	0.51	15
Anhui	2.00	24	0.49	19
Fujian	2.96	14	0.51	16
Jiangxi	3.77	9	1.02	9
Shandong	1.04	30	0.15	31
Henan	3.43	10	1.29	8
Hubei	2.61	17	0.72	11
Hunan	4.61	7	1.72	7
Guangdong	4.01	8	0.90	10
Guangxi	14.28	2	7.81	1
Hainan	2.17	21	0.62	13
Chongqing	12.58	4	4.10	6
Sichuan	21.42	1	4.71	2
Guizhou	13.20	3	4.53	4
Yunnan	11.79	5	4.62	3
Tibet	0.90	31	0.20	29
Shaanxi	2.86	15	0.52	14
Gansu	2.29	18	0.50	18
Qinghai	3.32	11	0.71	12
Ningxia	2.12	22	0.38	21
Xinjiang	9.50	6	4.32	5

^a^Excluding Hong Kong, Macao, and Taiwan regions

### Build models

We used the optimal subset regression method, set M = 4 (the formula of M ≤ INT(N/3), where N = 12, then M ≤ 4), to build regression equations, calculate the estimated value of the original sequence, and then calculate the CSC value of each model.

#### AIDS incidence models

Table 3 shows the four-time series MGFMs of AIDS incidence. We use each model formula to calculate the fitting values of AIDS incidence of each model from 2008 to 2019. The parameters of MAE, MAPE, MSE and RMSE of each AIDS incidence model can be viewed in Additional file 1: Table S1. By comparison, the R^2^ of each AIDS incidence model is relatively close: MAE, MAPE, MSE and RME4.

**Table 3:** Models of the MGFM of AIDS incidence

K	Regression equation	CSC	R	R^2^
1	$\mathrm{Y}=0.573+0.88254{x}_{1}$	11.6251	0.9782	0.9569
2	$\mathrm{Y}=0.567+1.41761{x}_{1}-0.52427{x}_{3}$	10.6892	0.9793	0.9590
3	$\mathrm{Y}=0.452+1.43702{x}_{1}+1.39078{x}_{2}-1.88978{x}_{3}$	9.0377	0.9819	0.9641
4	$\mathrm{Y}=0.447+1.43829{x}_{1}+1.46736{x}_{2}-1.96522{x}_{3}-0.10010{x}_{4}$	9.9969	0.9819	0.9641

**Fig. 4:**
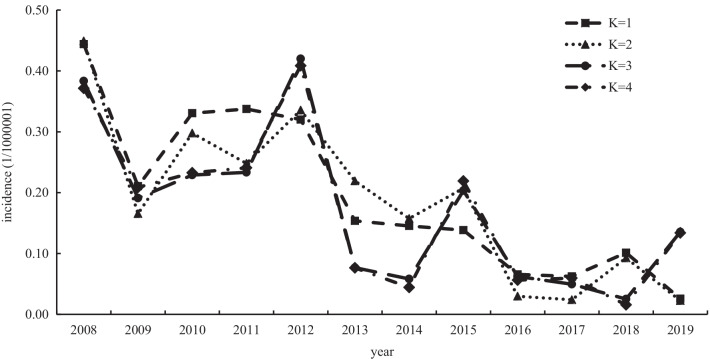
The absolute value distribution diagram of the difference between the fitting value and actual value of each prediction model of the AIDS incidence rate

To show the difference between the fitted value and the actual value of each AIDS incidence model more intuitively, we calculated the difference between the fitted value and the actual value of each AIDS incidence model and took the absolute value of the difference of each AIDS incidence model to draw Fig. 4. Figure 4 shows that the absolute value change of the residual error of the K = 1 and K = 2 AIDS incidence models is more stable, which shows that the fitting effect of the K = 1 and K = 2 AIDS incidence models is more reliable than that of the K = 3 and K = 4 AIDS incidence models. Finally, according to the principle of the CSC scoring method, combined with the parameters of the model and the results of Fig. 4, we selected the model with K = 1 as the optimal equation to predict the incidence of AIDS.

#### AIDS mortality models

Table 4 shows the four-time series MGFMs of AIDS mortality. Similarly, we use each model to calculate the fitting values of AIDS mortality of each model from 2008 to 2019. The parameters of MAE, MAPE, MSE and RMSE4 of each AIDS mortality model can be viewed in Additional file 1: Table S2. By comparison, the R^2^ of each AIDS mortality model was relatively close: MAE, MAPE, MSE and RMS.

**Table 4:** Models of the MGFM of AIDS mortality

K	Regression equation	CSC	R	R^2^
1	$\mathrm{Y}=0.090+0.857864{x}_{1}$	13.6804	0.9700	0.9409
2	$\mathrm{Y}=0.086+1.54683{x}_{1}-0.67634{x}_{3}$	15.0359	0.9715	0.9438
3	$\mathrm{Y}=0.050+1.55108{x}_{1}+0.94546{x}_{2}-1.58979{x}_{3}$	14.1185	0.9730	0.9467
4	$\mathrm{Y}=-0.092+1.36022{x}_{1}+1.13842{x}_{2}-1.59682{x}_{3}+0.16739{x}_{4}$	14.3683	0.9738	0.9483

**Fig. 5:**
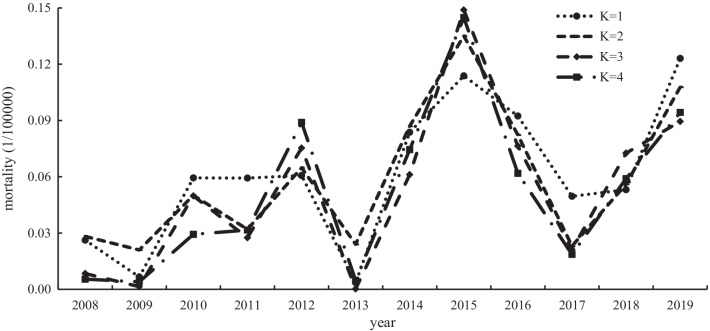
The absolute value distribution diagram of the difference between the fitting value and actual value of each prediction model of AIDS mortality

Similar to the AIDS incidence model, we calculated the difference between the fitted value and the actual value of each AIDS mortality model and drew Fig. 5 by taking the absolute value of the difference of each AIDS mortality model. Figure 5 shows that the absolute value change of the residual of AIDS mortality model with K = 2, K = 3 and K = 4 is more stable, it shows that the fitting effect of K = 2, K = 3 and K = 4 AIDS incidence models is more reliable than that of K = 1 AIDS mortality model. Finally, according to the principle of the CSC scoring method, combined with the parameters of the model and the results of Fig. 5, we selected the model with K = 2 as the optimal equation for the prediction of AIDS mortality.

### Prediction

Using the optimal equation selected above, the short-term prediction of the incidence (K = 1) and mortality (K = 2) of AIDS in China from 2020 to 2023 was made. The results show that the predicted values of AIDS incidence in China were 5.2816/100,000, 5.4581/100,000, 5.9670/100,000 and 6.3201/100,000, and the predicted values of AIDS mortality in China were 1.4294/100,000, 1.4502/100,000, 1.6111/100,000 and 1.7092/100,000, respectively. The results show that the incidence and mortality of AIDS in China will continue to rise.

## Discussion

At present, HIV is still a major global public health problem, which has claimed 36.3 million lives thus far [17]. The spread of AIDS is influenced by local political, economic, and cultural factors, which are often difficult to quantify [18, 19]. With the help of various technical means to establish an early warning mechanism for the HIV epidemic and predict the changing trends of AIDS in the future, public health departments could better prepare countermeasures in advance and effectively control the spread and prevalence of HIV. The MGFM can use the known finite time series data to build a prediction model with a good fitting effect by means of a mathematical model modelling method.

At present, the MGFM is widely used in disaster prediction, weather forecasting, preproduction of agricultural products and other disciplines [20, 21]. However, no related research has been found in the fields of medical management and public health. To the best of our knowledge, this study is the first to introduce the MGFM into the prediction of AIDS morbidity and mortality, and it verifies the applicability of the MGFM in the prediction of AIDS morbidity and mortality.

The results of the models constructed by our research showed that the determination coefficients of all models of AIDS incidence rate are above 0.95, and the determination coefficients of all models of AIDS mortality rate are above 0.94, and MAE, MAPE, MSE and RMSE of the two types of models are relatively close, indicating that the MGFM of AIDS incidence and mortality constructed by this study has good fitting effect and good accuracy. Therefore, MGFM can be used to predict the incidence and mortality of AIDS in China and can be used as a reliable method for disease prediction in the field of public health.

As shown in Table 1, the incidence of AIDS in China was relatively well controlled in 2013, 2014, 2016 and 2017, with an annual growth rate of less than 10%. However, in 2018 and 2019, the incidence showed a rebound trend, with an annual growth rate of more than 10%. From 2008 to 2019, the incidence rate of AIDS in China rose from 12 to 7th, and the mortality of AIDS in China from 2012 to 2019 remained 1st. AIDS has become one of the major infectious diseases in China, and the rising incidence and mortality rate showed that the epidemic is becoming increasingly serious, which needs to be given great attention by Chinese public health departments.

The top five areas of AIDS incidence in 2019 were Sichuan (21.42/100,000), Guangxi (14.28/100,000), Guizhou (13.20/100,000), Chongqing (12.58/100,000) and Yunnan (11.79/100,000). The top five areas of AIDS mortality are Guangxi (7.81/100,000), Sichuan (4.71/100,000), Yunnan (4.62/100,000), Guizhou (4.53/100,000) and Xinjiang (4.43/100,000). As shown in Figs. 2 and 3, these provinces were all located in western China. This result showed that the western region of China was the focal point of the AIDS epidemic, which was consistent with the research results of Wang et al. [22]. Studies have shown that, compared with eastern China, the economic development and cultural level in western China are relatively backward, and drug bootlegging exists in some areas [23, 24]. These were the key factors leading to the rapid spread of AIDS. The Chinese government should continue to increase investment in public health services, strengthen AIDS health education and control the spread of AIDS in western China [25].

The prediction results of our study showed that the incidence and mortality of AIDS in China will continue to rise from 2020 to 2023, which was similar to Hu et al. [26], Guo [27], and Xu et al. [12]. An in-depth analysis of our research showed that the reasons for the rising incidence and mortality of AIDS are as follows. First, with the market reform and opening up of the economy, followed by the rapid development of the regional economy, industrialization accelerated and the population mobility increased, thus accelerating the spread of AIDS [27]. Second, influenced by the concept of sexual liberation, some people ignored sexual morality and believed in sexual openness [27]. Third, unclean sexual behaviours such as unprotected sex and men who have sex with men are very serious at present [28]. Fourth, this may be related to the importance attached to the HIV/AIDS epidemic in China. The Chinese direct reporting system and related monitoring networks are constantly improving and maturing, which plays an extremely important role in finding hidden infected people [29].

To cope with the rising incidence and mortality of AIDS, Chinese public health departments should constantly promote AIDS prevention and treatment. First, Chinese health departments should increase publicity and education for Chinese citizens to popularize the main transmission routes of AIDS. Second, investment in research and development of AIDS vaccines should be increased, clinical treatment methods should be improved and early screening efforts should be strengthened as early as possible to identify patients in the incubation period. Finally, the government should constantly improve measures such as humanistic care for AIDS patients, psychological counselling and educational guidance [30–32].

## Conclusion

In summary, our research proved that the MGFM had a high fitting accuracy in the prediction of AIDS morbidity and mortality in China and can be applied to the prediction of AIDS mortality and morbidity in China. The research also found that the western region of China was the main area of the AIDS epidemic in the country and needs to be highly attended to by the Chinese health department, which can formulate intervention policies according to local conditions.

## Limitations

There were some limitations in this research. First, this research only predicted the epidemic trend of AIDS from the perspective of macroscopic data and failed to analyse the influencing factors of the occurrence and spread of the disease. Tt is impossible to analyse the cultural, educational, and economic factors related to the AIDS epidemic in China. Second, the incidence of AIDS is related to region and season. In this research, we did not establish the epidemic model of monthly and quarterly data, which cannot reflect the changes in monthly or quarterly AIDS incidence and mortality in China. This will be the key research content of our team in the future. Finally, this study only discussed the prediction of AIDS incidence and mortality in China from a theoretical perspective. In fact, the AIDS epidemic may be influenced by various factors, and there may still be a gap between our research and the actual situation.

## Supplementary Information

**Additional file 1.** **Table S1** Parameters of four MGFMs of AIDS incidence. **Table S2** Parameters of four MGFMs of AIDS mortality.

## Data Availability

The data in this paper are from the *China Health Statistics Yearbook 2020*, which was published by Peking Union Medical College Press in 2020. All data analysed during this study are included in this published article.

## Abbreviations

AIDS: Acquired immune deficiency syndrome
HIV: Human immunodeficiency virus
CDC: Center for Disease Control
MGFM: Mean generation function model
ARIMA: Autoregressive integrated moving average
MAE: Mean absolute error
MAPE: Mean absolute percentage error
MSE: Mean squared error
RMSE: Root mean square error
