# Supplementary material for: Mean generation function model in AIDS epidemic estimation
Source: BMC Med Inform Decis Mak. 2022 Apr 16;22:104. doi: 10.1186/s12911-022-01825-6 (PMC9013215; doi:10.1186/s12911-022-01825-6)
Supplement: Supplementary file 1 — Additional file 1. Table S1 Parameters of four MGFMs of AIDS incidence. Table S2 Parameters of four MGFMs of AIDS mortality. [file 12911_2022_1825_MOESM1_ESM.docx]

Supplement

Table 1 Parameters of four MGFMs of AIDS incidence

| K | MAE | MAPE | MSE | RMSE |
| --- | --- | --- | --- | --- |
| 1 | 0.1946 | 8.9970 | 0.6501 | 0.8063 |
| 2 | 0.1874 | 8.6551 | 0.6183 | 0.7863 |
| 3 | 0.1723 | 7.8027 | 0.5427 | 0.7367 |
| 4 | 0.1723 | 7.8005 | 0.5413 | 0.7357 |

Table 2 Parameters of four MGFMs of AIDS mortality

| K | MAE | MAPE | MSE | RMSE |
| --- | --- | --- | --- | --- |
| 1 | 0.0610 | 6.4608 | 0.0050 | 0.0709 |
| 2 | 0.0593 | 6.4465 | 0.0048 | 0.0692 |
| 3 | 0.0527 | 5.3983 | 0.0045 | 0.0674 |
| 4 | 0.0514 | 5.2612 | 0.0044 | 0.0664 |
